# Correlation between changes of amino acid spectrum and alopecia in patients with obesity undergoing bariatric surgery: a prospective cohort study

**DOI:** 10.3389/fnut.2025.1618630

**Published:** 2025-08-26

**Authors:** Tingwen Wang, Jiaqing Huang, Xin Sun, Hai Zhao, Jun Bao, Xuehui Chu, Xiaojie Bian

**Affiliations:** ^1^Department of Pharmacy, Nanjing Drum Tower Hospital, Affiliated Hospital of Medical School, Nanjing University, Nanjing, China; ^2^Department of Pharmacy, Nanjing Drum Tower Hospital, School of Basic Medicine and Clinical Pharmacy, China Pharmaceutical University, Nanjing, China; ^3^Department of Pharmacy, Nanjing Women and Children’s Healthcare Hospital, Women’s Hospital of Nanjing Medical University, Nanjing, China; ^4^Key Laboratory of Child Development and Learning Science of Ministry of Education of China, School of Biological Sciences and Medical Engineering, Southeast University, Nanjing, China; ^5^Department of Dermatology, Nanjing Drum Tower Hospital, Affiliated Hospital of Medical School, Nanjing University, Nanjing, China; ^6^Department of Pancreatic and Metabolic Surgery, Nanjing Drum Tower Hospital, Affiliated Hospital of Medical School, Nanjing University, Nanjing, China

**Keywords:** amino acids, bariatric surgery, alopecia, leucine, obesity

## Abstract

**Purpose:**

To investigate the association between serum amino acid changes and alopecia in patients with obesity before and after bariatric surgery, and identify risk factors for postoperative alopecia.

**Methods:**

This prospective cohort study (ChiCTR2300074104) included patients with obesity undergoing laparoscopic sleeve gastrectomy (LSG), categorized into non-mild alopecia (NM group, *n* = 24) and moderate–severe alopecia (MS group, *n* = 43) groups. Clinical data and serum amino acid concentrations were analyzed to: (1) compare preoperative and postoperative amino acid profiles; (2) evaluate amino acid differences between groups at various timepoints and correlate with alopecia severity; (3) perform logistic regression to identify alopecia risk factors.

**Results:**

Among 67 patients analyzed, LSG significantly decreased serum concentrations of arginine, alanine, threonine, glutamic acid, branched-chain amino acids (valine, isoleucine, leucine), and aromatic amino acids (tyrosine, phenylalanine, tryptophan) (*p* < 0.05), while serine and glycine increased (*p* < 0.05). At 3 months postoperatively, leucine levels were higher in the MS group compared to the NM group. Spearman’s correlation analysis revealed threonine, *γ*-aminobutyric acid, and leucine levels were associated with alopecia severity (*p* < 0.05). Logistic regression identified serum leucine as an independent risk factor for postoperative alopecia (OR = 1.119, 95% CI 1.006-1.245, *p* = 0.038).

**Conclusion:**

LSG surgery alters the serum amino acid profile in patients with obesity, and serum leucine concentration at 3 months postoperatively is an influential factor in postoperative alopecia.

## 1 Introduction

With the change in people’s diet structure and lifestyle, the obese population is increasing. The World Health Organization estimates that in 2016, there were over 1.9 billion adults worldwide who were overweight, of which over 650 million were obese. The prevalence of obesity has exceeded 30% in some developed nations, and by 2030, there are expected to be over 366 million patients with obesity (1). Obesity-related complications such as cardiovascular disease, type 2 diabetes, and cancer (2, 3) will turn out to be a major challenge for global healthcare. Bariatric surgery should be considered for patients with severe obesity with body mass index (BMI) ≥ 40 kg/m^2^ or BMI ≥ 35.0 kg/m^2^ with associated complications (4), which is effective in achieving weight loss, improving the outcome of adverse cardiovascular events and decreasing mortality (5, 6).

Because it is safe and simple to do, laparoscopic gastric sleeve surgery (LSG) is one of the restrictive surgical techniques most frequently used for patients who are moderately to severely obese, which reduces the absorption of gastric volume to promote weight loss (7). However, the change in gastric volume as a result of LSG may cause patients to feel less hungry, reduced dietary intake leading to insufficient supplementation of protein intake, and patients to suffer from malnutrition and related postoperative complications (8). Alopecia is a common postoperative complication after LSG that causes great psychological stress to the patient, the exact cause of which is still unclear. Amino acids are essential for protein synthesis and hair follicle function, their deficiency or imbalance may contribute to alopecia, and one study showed differences in serum amino acid concentrations between patients with alopecia and the healthy population (9). Due to modifications in body metabolism, patients with obesity blood amino acid profiles alter post-surgery. A cross-sectional investigation revealed that the concentrations of branched-chain amino acids in serum were higher in patients with obesity and significantly lower following sleeve gastrectomy (10). In this study, we compared the changes in preoperative and postoperative amino acid profiles of patients with obesity proposed to undergo LSG surgery and examined the amino acid levels at different time points in the postoperative alopecia and non-alopecia groups of subjects, respectively, to find biomarkers with a strong correlation with alopecia.

## 2 Materials and methods

### 2.1 Study population

This was a prospective, single-center, observational cohort study. The subjects were recruited from the Department of Gastrointestinal Surgery of a tertiary general hospital in Nanjing for LSG from August 2021 to February 2022 The inclusion criteria were as follows: (1) The patients were 18-60 years old; (2) the procedure was LSG; (3) the BMI was >30 kg/m^2^; (4) the patients voluntarily signed an informed consent form and adhered to the requirements of the study protocol, and were able to complete a minimum of 3 months of postoperative follow-up. Exclusion criteria were as follows: (1) history of severe hair loss before surgery [normal hair loss is defined as no more than 100 hairs per day (11)]; (2) individuals who declined to participate in the follow-up or left the trial; (3) those with severe renal, hepatic, and cardiac impairment; (4) two or more operations were performed during hospitalization; (5) serious complications or even death occurred after operation; (5) Known refractory metabolic diseases, such as poorly controlled diabetes mellitus (e.g., fasting blood glucose ≥10 mmol/L), hyperthyroidism, hypothyroidism; (6) Pregnant or lactating women.

Patients were categorized into non-mild alopecia group (NM group) or moderate–severe alopecia group (MS group) based on skin microscopy examination diagnosis during the 3-month postoperative outpatient follow-up. The study was registered with the Chinese clinical trial registry^1^, registration numbers: ChiCTR2300074104. The study was approved by the Ethics Committee of Nanjing Drum Tower Hospital, Affiliated Hospital of Medical School, Nanjing University (2021-334-02). Study subjects signed an informed consent form.

### 2.2 Study methods

#### 2.2.1 Data collection

Patients gender, age, diagnosis, BMI and other clinical information were collected from Hospital Information System. Enrolled patients completed postoperative outpatient follow-up at months 1 and 3 during the study cycle. According to the American Society for Metabolic and Bariatric Surgery (4), the patient’s post-discharge protein supplementation regimen was: whey protein (Swisse Wellness Pty Ltd) 60 g/day. The caloric intake protocol: 400 kcal/day for the first week; 600-800 kcal/day during weeks 3–4; and 1,200–1500 kcal/day for 2–6 months.

Postoperative follow-up is conducted by our hospital’s multidisciplinary weight loss and metabolism team. This team provides postoperative dietary education and a dietary guidance sheet to each enrolled patient. During each follow-up visit, the patient’s dietary status is assessed, and adjustments are made as necessary. Additionally, patient and family education is enhanced to improve patient adherence to treatment and self-management abilities, ensuring the implementation of the protocol and the effectiveness of weight loss.

#### 2.2.2 Alopecia diagnostic criteria

Dermatologists from our hospital examined the patient at the time of admission and 3 months after surgery utilizing skin microscope (Jiangsu Jieda Technology Development Co., Ltd., Model JD-801) hair analysis testing technologies to assess hair growth condition. The occurrence and amount of alopecia were determined using the Ludwig classification for female alopecia (12) or the Hamilton-Norwood (H-N) classification system for male alopecia (11). Using a seven-point grading scale (13) (−3: significant decrease, −2: moderate decrease, −1: slight decrease,0: no change, +1: slight increase, +2: moderate increase, +3: significant increase) to compare baseline macroscopic images to those taken 3 months after surgery to assess the hair loss problem in conjunction with the patient’s symptoms.

#### 2.2.3 Blood sample collection and serum amino acid testing

1.5 mL of serum samples remaining after the subjects’ preoperative, 1- and 3-month postoperative routine examinations were collected in 2 mL centrifuge tubes and stored at −80 °C. The serum samples were thawed before the assay and the concentrations of 17 amino acids by High-Performance Liquid Chromatography with o-phthalaldehyde Online Pre-column Derivatization (14), including taurine, serine, glutamine, glycine, threonine, aspartic acid, arginine, alanine, glutamic acid, *γ*-aminobutyric acid (GABA), tyrosine, valine, phenylalanine, isoleucine, leucine, methionine and tryptophan. The serum amino acid concentrations of the different species were calculated using a calibrated working curve at the completion of the experiments.

Experimental equipment: LC-20 AD Ultra-high Performance Liquid Chromatograph, L-ECD 6A Electrochemical Detector (Shimadzu Co., Ltd., Japan) and VP-ODS column (4.6 × 150 mm, 5 μm, Shimadzu Co., Ltd., Japan).

Chemicals and Reagents: HPLC grade methanol and working standards (taurine, serine, glutamine, glycine, threonine, aspartic acid, arginine, alanine, glutamic acid, GABA, tyrosine, valine, phenylalanine, isoleucine, leucine, methionine and tryptophan) were purchased from Sigma-Aldrich (Milano, Italy). Sodium tetraborate, sodium sulfite, o-phthalaldehyde, sodium hydroxide, hydrochloric acid, citric acid and perchloric acid from Sinopharm Chemical ReagentCo., Ltd.(Shanghai, China).

There are two types of mobile phases: the weak retention system mobile phase consists of acetonitrile 130 mL, methanol 100 mL, water 1770 mL, citric acid 22.8 g, NaH_2_PO_4_ 21.9 g and anhydrous EDTA 0.15 g. Strongly retained mobile phase: methanol 640 mL, water 1,360 mL, KH_2_PO_4_ 8.84 g and anhydrous EDTA 0.05 g. The mobile phase was filtered by 0.45 μm microporous filtration organic membrane and degassed by ultrasonication. The detection potential, mobile phase flow rate, injection volume, and column temperature were 0.7 V, 1.0 mL/min, 20 μL, 30 °C.

### 2.3 Sample size calculation

The primary endpoint of this study was serum amino acid concentration at 3 months postoperatively, and there are no relevant studies that provide serum amino acid concentrations in NM groups versus MS groups. We followed guidelines for minimum sample sizes for pilot studies (15) which recommends the inclusion of 10 ~ 40 people per group. In conjunction with the practicalities of this study, 20 participants were included in each group at least.

### 2.4 Statistical analysis

Continuous variables are expressed as mean±SD or medians with interquartile ranges. Between-group comparisons at baseline in continuous data were tested with the independent-sample t-test or Mann–Whitney U test, in which the categorical variables were tested with the χ^2^ test or Fisher exact test, and expressed as a rate (%). For comparisons of amino acid concentrations at different time points, using analysis of variance for repeated measurements or generalized estimating equation (GEE). Correlation analysis was performed using Spearman’s method. Logistic regression analysis was used to screen for independent influences affecting patients’ postoperative alopecia. The level of significance was set as a 2-sided *p* value less than 0.05. All analyses were conducted with SPSS version 26.0 (SPSS Inc), and graphing with Graphpad 9.0.

## 3 Results

### 3.1 Patient characteristics

**Figure 1:**
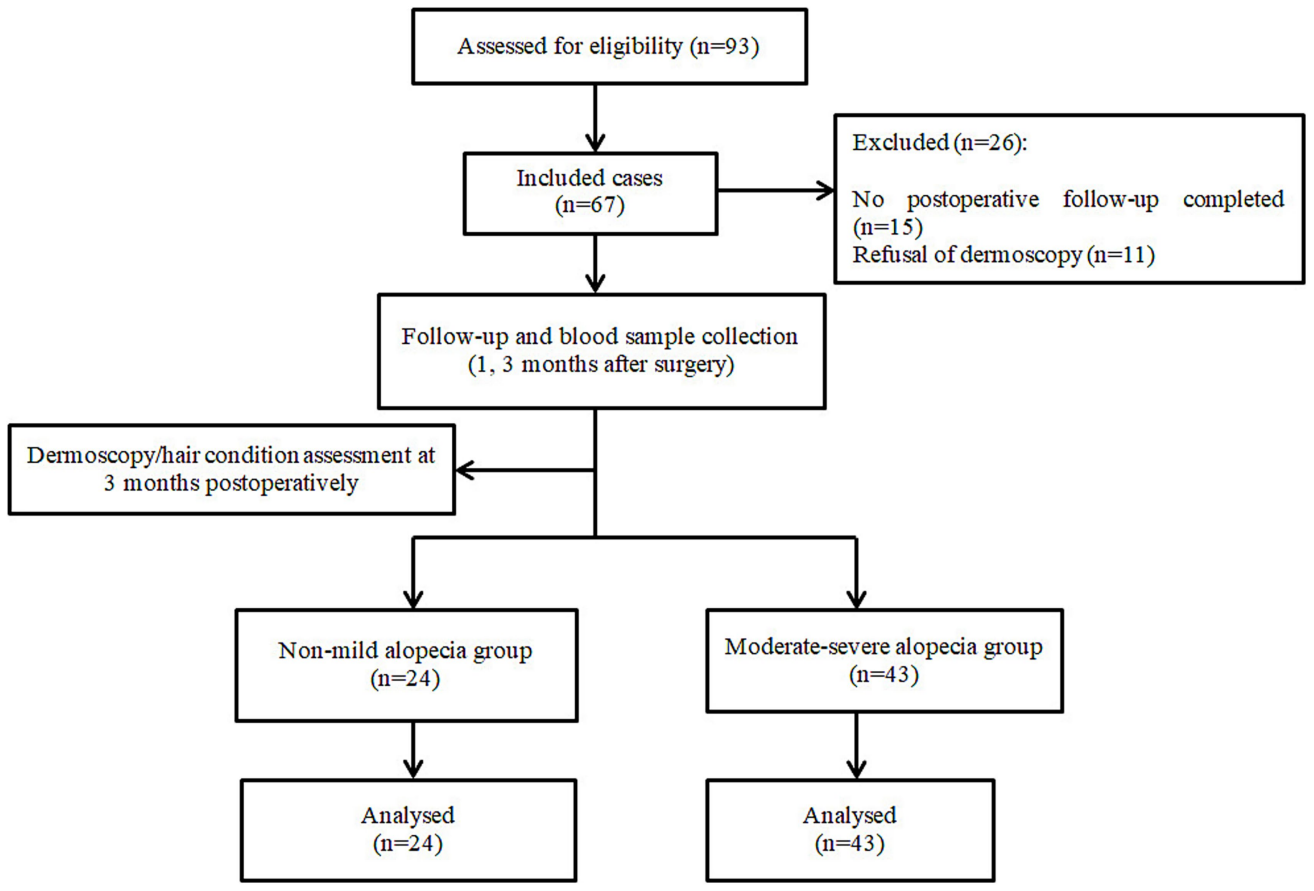
Flow chart of the study.

A total of 93 LSG patients were enrolled in the pre-study period, 26 patients who did not complete 3 months of outpatient follow-up and refused to sign the informed consent were excluded, and 67 patients were finally included, and the flow chart of the study is shown in Figure 1. To assess potential selection bias, we compared the baseline characteristics of included and excluded patients. The results showed no statistically significant differences in baseline characteristics (*p* > 0.05), indicating that the exclusion of patients did not significantly affect the representativeness of the study population.

There were 24 males (35.82%) and 43 females (64.18%), with a mean age of 32.59 ± 7.90 years, a mean BMI of 37.92 ± 6.24 kg/m^2^. A total of 43 patients with moderate to severe alopecia were observed in the postoperative period, with an incidence of alopecia of 64.18%, a male alopecia ratio of 14/24, and a female alopecia ratio of 29/43. The time of onset of alopecia was 2.88 ± 0.87 months postoperatively. The patients’ baseline demographic and clinical characteristics data are detailed in Table 1, and no significant differences between the MS group and the NM group were noted (*p* > 0.05). There was no statistically significant difference in weight loss between the two groups at one and 3 months postoperatively.

**Table 1:** Baseline characteristics of participants.

Characteristics	MS group (*n* = 43)	NM group (*n* = 24)	*p*-value
Age (years)	29 (25, 39)	34 (30, 40)	0.064
Male (*n*, %)	14 (32.56)	10 (41.67)	0.456
Height (cm)	100 (90, 128)	105.5 (93.25, 132)	0.433
Weight (kg)	168 (162, 175)	168 (163.25, 176)	0.943
BMI (kg/m^2^)	37.39 ± 6.16	38.81 ± 6.39	0.374
Waist (cm)	117.15 ± 13.93	118.48 ± 17.19	0.732
Hip (cm)	121.23 ± 11.28	121.77 ± 11.29	0.852
WHR	0.97 (0.92, 1.02)	0.96 (0.93, 1.02)	0.748
SBP (mmHg)	145 (128, 152)	144.5 (128, 151)	0.896
DBP (mmHg)	90.14 ± 11.33	91 ± 8.07	0.72
Course of obesity (years)	5 (3, 10)	6.5 (3, 13)	0.389
Types of obesity-related comorbidities (*n*)	5 (3, 6)	4.5 (3.2, 6)	0.76
ALT (U/L)	35 (24.3, 59.0)	38.5 (23.0, 67.7)	0.137
AST (U/L)	28.17 ± 5.30	26.36 ± 4.72	0.49
TG (mmol/L)	1.8 ± 0.6	1.6 ± 0.7	0.615
TC (mmol/L)	4.8 ± 1.3	4.7 ± 1.2	0.439
HDL-C (mmol/L)	1.0 ± 0.3	1.1 ± 0.5	0.465
LDL-C (mmol/L)	3.1 ± 1.2	2.9 ± 0.8	0.18
HbA_1_C (mmol/mol)	39.06 ± 8.06	35.34 ± 5.58	0.08
Hypoglycemia (mmol/L)	5.7 ± 1.9	5.4 ± 1.4	0.323
Uric acid (μmol/L)	339.23 ± 23.5	350.20 ± 21.3	0.127

Normally distributed variables were expressed as mean ± standard deviation; skewed distributed variables as median and interquartile range; *p*-value for differences between two groups were calculated with Mann–Whitney U test for skewed distributed variables and *T*-test for normal distributed and χ^2^ test for categorical variables; BMI, body mass index; WHR, Waist-to-Hip Ratio; SBP, systolic blood pressure; DBP, diastolic blood pressure; ALT, glutamic-pyruvic transaminase; AST, glutamic oxaloacetic transaminase; TG, triglyceride; TC, total cholesterol; HDL-C, High-density lipoprotein cholesterol; LDL-C, Low-density lipoprotein cholesterol; HbA1C, glycosylated hemoglobin; NM, non-mild alopecia group; MS, moderate–severe alopecia group.

When the amino acid concentrations of the patients in the NM group and the MS group were compared, the analysis showed that there was no statistically significant difference between the two groups’ preoperative amino acid concentrations (*p* > 0.05). Supplementary Table 1 displays the preoperative amino acid levels of the patients in the two groups.

### 3.2 Comparative analysis of amino acid levels in patients with obesity before and after bariatric surgery

**Figure 2:**
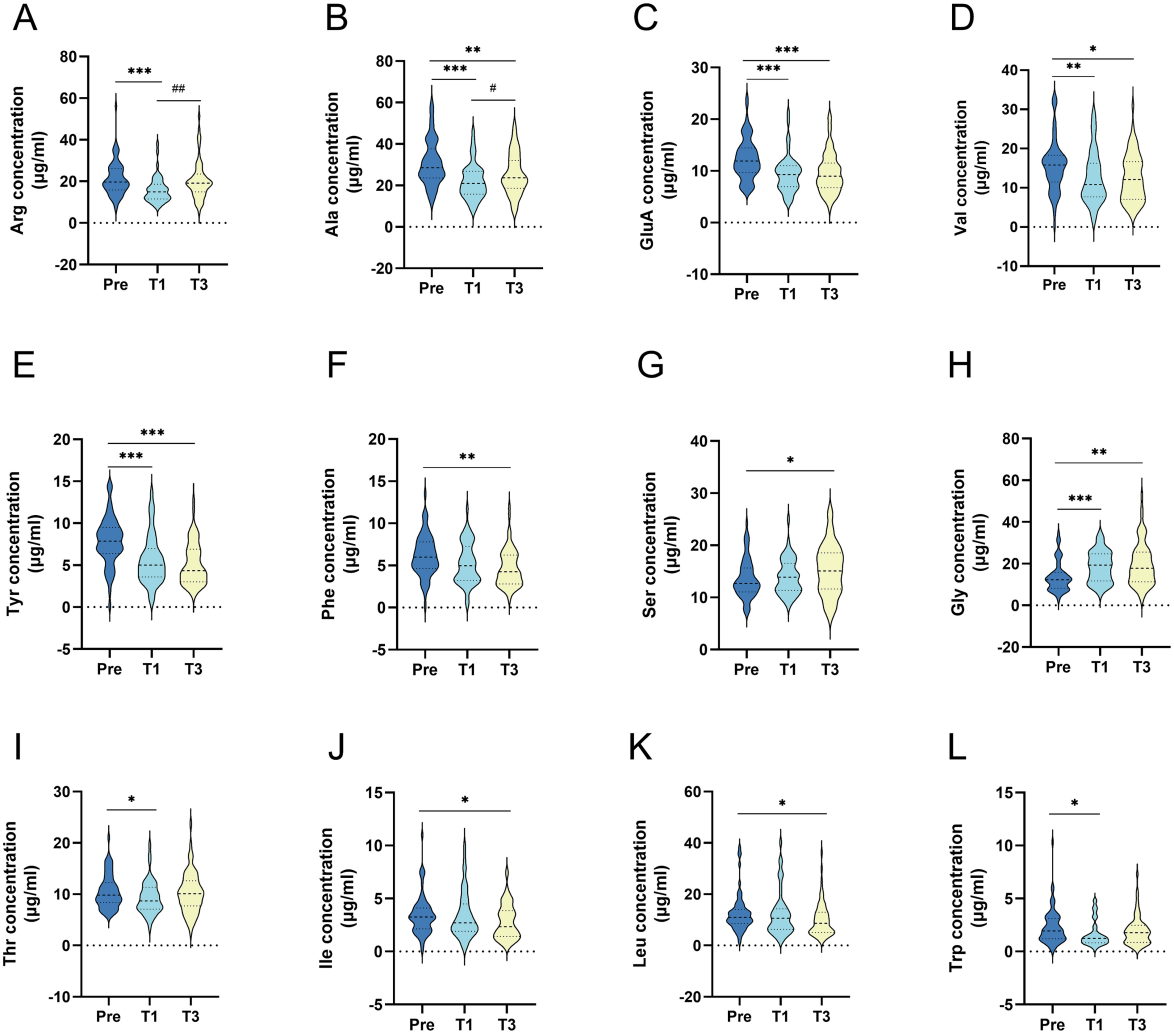
Comparison of serum amino acid concentrations before and after surgery in all patients **(A)** Arginine; **(B)** Alanine; **(C)** Glutamic acid; **(D)** Valine; **(E)** Tyrosine; **(F)** phenylalanine; **(G)** Serine; **(H)** Glycine; **(I)** Threonine; **(J)** isoleucine; **(K)** leucine; **(L)** Tryptophan. All data were presented as median (inter quartile range); concentration units are μg/ml; *p*-Value: generalized estimating equation analysis; Pre: preoperative; T1: 1 month after surgery; T3: 3 months after surgery; (*) statistical difference between pre and after surgery: *p<0.05, **p<0.01, ***p<0.001; (#) statistical difference wtih one mouth postoperatively. #*p*<0.05, ##*p*<0.01, ###*p*<0.001.

Comparative analysis of serum amino acid concentration data for all subjects during the preoperative and follow-up periods using the GEE method is shown in Supplementary Table 2, and the positive results are represented in Figure 2. The findings revealed that the amino acid profile of patients with obesity changed following LSG, with serum concentrations of most amino acids decreasing postoperatively compared to preoperative levels. Arginine, alanine, threonine, glutamic acid, branched-chain amino acids (BCAAs, valine, isoleucine and leucine), and aromatic amino acids (AAAs, tyrosine, phenylalanine, tryptophan) showed significant decreases in serum concentrations postoperatively (*p* < 0.05); serine and glycine showed significant increases postoperatively (*p* < 0.05); and the concentrations of the other amino acids remained essentially unchanged during the follow-up period.

### 3.3 Comparative analysis of amino acid levels at different time points between-group

The amino acid concentrations of MS and NM patients were compared at different time points (preoperative, 1 and 3 months postoperative), and the results are shown in Table 2. At 3 months postoperative, the serum leucine concentration was significantly higher in the MS group than in the NM group (9.88 [5.65, 14.95] vs. 6.30 [4.68, 12.02], *p* < 0.05).

**Table 2:** Compassion of plasma amino acid concentration between two groups at the 1, 3 months of postoperation.

Amino acids	Group	Pre	T1	T3
Taurine	MS group	12.06 (9.38, 17.16)	15.01 (6.17, 25.36)	19.57 (9.65, 25.47)
	NM group	15.01 (9.92, 22.78)	12.06 (7.78, 20.11)	14.21 (23.59, 9.12)
	*p*-value	0.593	0.539	0.183
Serine	MS group	12.95 (11.08, 15.75)	14.22 (11.52, 17.16)	14.95 (11.08, 19.63)
	NM group	12.55 (10.81, 15.22)	12.68 (11.01, 15.55)	15.75 (11.98, 18.39)
	*p*-value	0.984	0.52	0.691
Glutamine	MS group	58.09 (49.39, 64.70)	53.13 (41.43, 63.22)	54.96 (44.35, 68.52)
	NM group	57.39 (44.70, 62.09)	51.13 (48.26, 66.78)	57.13 (51.09, 69.35)
	*p*-value	0.803	0.814	0.53
Glycine	MS group	11.80 (7.71, 15.90)	19.40 (10.42, 24.88)	18.07 (10.36, 25.78)
	NM group	12.28 (10.12, 15.66)	16.87 (13.13, 24.46)	15.30 (12.05, 24.76)
	*p*-value	0.389	0.476	0.536
Threonine	MS group	10.10 (8.42, 12.34)	8.56 (7.05, 11.01)	9.68 (6.31, 11.36)
	NM group	9.26 (7.71, 11.50)	9.54 (7.08, 12.34)	10.87 (8.70, 13.67)
	*p*-value	0.514	0.714	0.066
Aspartic	MS group	3.88 (3.01, 5.44)	3.30 (2.33, 4.08)	3.69 (2.72, 6.02)
	NM group	4.85 (3.69, 6.80)	3.50 (2.91, 4.37)	3.69 (2.33, 6.75)
	*p*-value	0.181	0.27	0.633
Arginine	MS group	20.05 (17.25, 25.18)	15.15 (11.42, 19.06)	18.88 (12.99, 23.78)
	NM group	17.48 (13.52, 26.10)	14.45 (11.54, 18.53)	19.35 (15.39, 22.84)
	*p*-value	0.551	0.9	0.906
Alanine	MS group	27.78 (23.69, 39.73)	19.13 (15.70, 27.73)	23.27 (17.30, 32.08)
	NM group	31.45 (23.27, 37.00)	21.70 (16.40, 25.42)	23.85 (21.83, 32.78)
	*p*-value	0.735	0.804	0.621
Glutamic acid	MS group	11.23 (9.43, 14.07)	9.81 (7.07, 11.04)	8.38 (6.44, 10.93)
	NM group	12.73 (10.93, 16.92)	8.83 (6.14, 11.00)	10.03 (7.82, 13.47)
	*p*-value	0.273	0.277	0.32
GABA	MS group	1.99 (1.18, 2.41)	1.21 (0.97, 1.66)	1.57 (1.09, 1.93)
	NM group	1.33 (0.56, 2.53)	1.45 (0.84, 2.05)	0.99 (0.77, 1.42)
	*p*-value	0.299	0.42	0.699
Tyrosine	MS group	7.99 (6.30, 9.28)	4.95 (3.07, 7.11)	4.61 (3.52, 7.55)
	NM group	7.86 (6.44, 10.21)	5.56 (4.03, 7.10)	4.33 (2.83, 6.13)
	*p*-value	0.839	0.373	0.11
Valine	MS group	14.90 (11.40, 17.04)	10.10 (6.97, 16.78)	12.74 (8.15, 17.33)
	NM group	17.36 (11.21, 20.26)	11.71 (8.29, 15.96)	9.81 (6.24, 16.29)
	*p*-value	0.939	0.89	0.051
Phenylalanine	MS group	6.04 (4.67, 7.91)	4.72 (3.16, 7.77)	4.33 (3.13, 6.32)
	NM group	5.65 (4.42, 7.72)	5.10 (3.30, 6.50)	3.78 (2.69, 5.37)
	*p*-value	0.772	0.999	0.104
Isoleucine	MS group	3.13 (2.10, 3.72)	2.50 (1.70, 4.53)	2.98 (1.57, 3.99)
	NM group	3.95 (2.14, 4.50)	2.85 (2.35, 4.61)	1.93 (1.14, 3.39)
	*p*-value	0.9	0.99	0.052
Leucine	MS group	10.53 (8.60, 13.62)	9.63 (5.48, 15.53)	9.88 (5.65, 14.95)
	NM group	11.81 (7.64, 16.81)	10.96 (7.22, 13.29)	6.30 (4.68, 12.02)
	*p*-value	0.726	0.452	0.020*
Methionine	MS group	0.48 (0.41, 0.50)	0.48 (0.24, 0.66)	0.48 (0.24, 0.72)
	NM group	0.48 (0.28, 0.78)	0.48 (0.24, 0.61)	0.28 (0.24, 0.48)
	*p*-value	0.943	0.958	0.25
Tryptophan	MS group	1.86 (1.20, 3.25)	1.24 (0.83, 1.75)	1.79 (0.97, 2.62)
	NM group	2.10 (1.18, 3.03)	1.10 (0.83, 2.24)	1.65 (0.83, 2.06)
	*p*-value	0.785	0.752	0.307

All data were presented as median (inter quartile range); concentration units are μg/ml; *p*-Value: generalized estimating equation analysis; * and Bold values indicate significant difference between the two groups (*p* < 0.05). NM, non-mild alopecia; MS, moderate–severe alopecia. Pre, preoperative; T1, 1month after surgery; T3, 3 months after surgery.

### 3.4 Analysis of the correlation between amino acid levels and the occurrence of alopecia

#### 3.4.1 All patients at 1 month postoperatively

**Figure 3:**
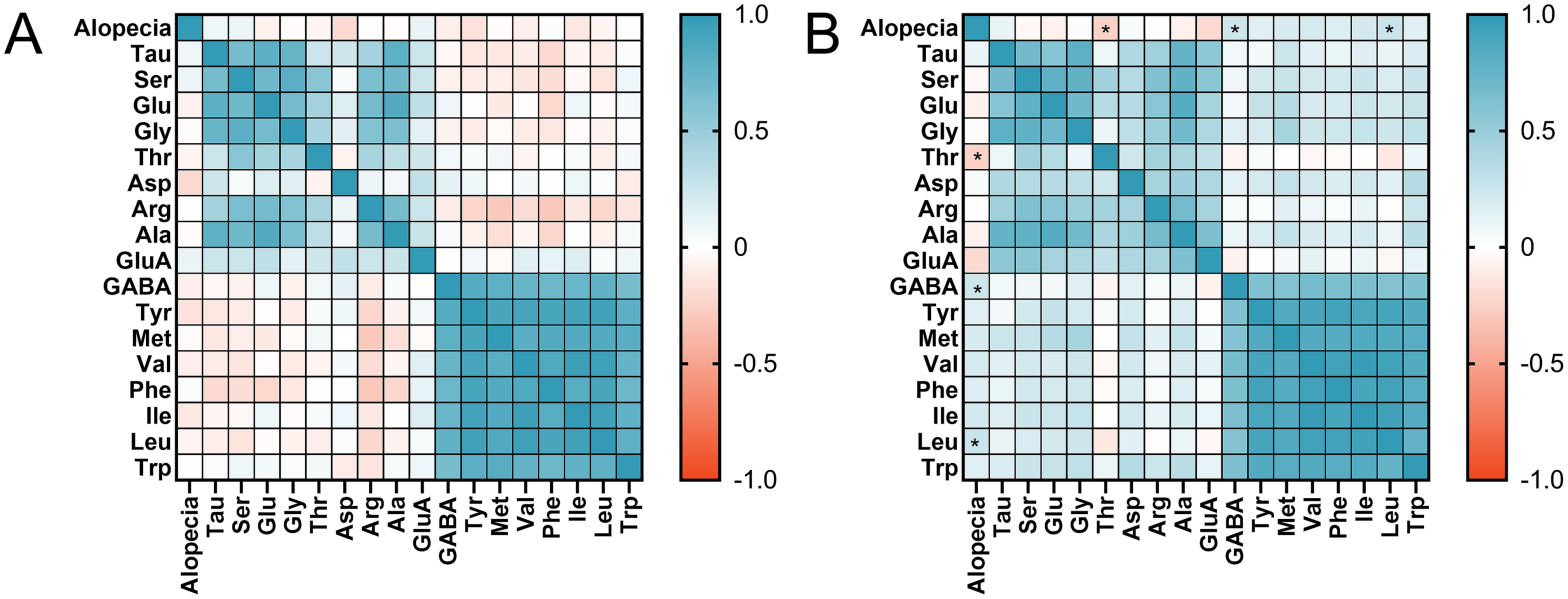
Heat map of the relationship between alopecia and amino acid levels: **(A)** at 1 month postoperatively; **(B)** at 3 month postoperatively. *p*-value from Spearman’s correlation analysis. Tau, taurine; Ser, serine; Glu, glutamine; Gly, glycine; Thr, threonine; Asp, aspartic acid; Arg, arginine; Ala, alanine; GluA, glutamic acid; GABA, *γ*-Aminobutyric acid; Tyr, tyrosine; Val, valine; Phe, phenylalanine; Ile, isoleucine; Leu, leucine; Met, methionine; Trp, Tryptophan.* *p*<0.05.

Spearman’s correlation coefficient ranges from [−1, 1], with less than 0 indicating negative correlation and more than 0 indicating positive correlation. Correlation coefficients closer to 0 are weaker, and correlation is only significant at *p* < 0.05. As demonstrated in Figure 3A, Spearman’s correlation study of amino acid content at 1 month postoperatively with the occurrence of alopecia revealed no significant link.

#### 3.4.2 All patients at 3 month postoperatively

The findings of Spearman’s correlation analysis revealed that the levels of threonine (*r* = −0.248, *p* = 0.043), GABA (*r* = 0.247, *p* = 0.044), and leucine (*r* = 0.263, *p* = 0.031) at 3 months postoperatively had an association with alopecia, as illustrated in Figure 3B. At 3 months postoperatively, threonine concentrations were negatively connected with alopecia, whereas GABA and leucine concentrations were positively correlated with alopecia occurrence.

### 3.5 Independent influences on alopecia in bariatric surgery patients

Whether alopecia occurred after bariatric surgery was used as the dependent variable. The indicators with statistically significant differences between the two groups in the above analysis and amino acids with significant correlation with postoperative alopecia (serum GABA, leucine, and threonine concentrations at 3 months postoperatively) were used as independent variables for logistic regression analysis using the forward stepwise method. As demonstrated in Table 3, the findings indicated that serum leucine was an independent risk factor (*p* < 0.05) for the development of alopecia in individuals 3 months following surgery.

**Table 3:** Logistic regression analysis of risk factors for hair loss in patients 3 month after weight loss metabolic surgery.

Variables	*β*	S. E.	Wald	*p*-value	OR	95% CI for OR	
						Lower	Upper
Leu	0.112	0.054	4.306	**0.038**	1.119	1.006	1.245
Constant	−0.459	0.532	0.743	0.389			

Bold values indicate significant difference between the two groups (*p* < 0.05).

## 4 Discussion

This single-center prospective cohort study classified patients into NM and MS groups based on 3-month postoperative dermatoscopic findings and compared serum amino acid concentrations preoperatively and at 1 and 3 months post-LSG to identify hair loss-related factors. To assess potential selection bias, we compared baseline characteristics between included (*n* = 67) and excluded (*n* = 26) patients, finding no significant differences (*p* > 0.05), indicating minimal impact on representativeness.

### 4.1 Analysis of serum amino acid concentration changes before and after LSG in obese patients

Our study observed decreased postoperative serum concentrations of most amino acids in obese patients following LSG. Significant reductions occurred in threonine, alanine, arginine, glutamic acid, branched-chain amino acids (BCAAs: valine, isoleucine, leucine), and aromatic amino acids (AAAs: tyrosine, phenylalanine, tryptophan), while serine and glycine showed significant increases (16, 17).

### 4.2 Mechanistic analysis of amino acid concentration changes before and after LSG

Threonine reduction aligns with previous studies (16, 17), potentially due to decreased postoperative protein intake, though the mechanism remains unclear. Alanine reduction may result from enhanced cellular uptake and glucose-alanine cycling (18–20). While our findings of decreased arginine levels align with increased metabolism (21–23), contrasting reports exist (19), warranting further investigation. Postoperative glutamate reductions may relate to gut microbiota changes affecting synthesis (21).

Elevated preoperative BCAAs, associated with obesity and insulin resistance (24, 25), decreased significantly postoperatively, consistent with prior findings (26, 27). Reductions in leucine (28) correlated with improved metabolic parameters, including insulin resistance and glycemic control (21). The decline in BCAAs reflects restored insulin function and reduced protein hydrolysis (10, 29). The reason for the decrease in the concentration of BCAAs in the postoperative period is related to the improvement of insulin resistance and the restoration of insulin’s ability to inhibit protein hydrolysis in patients with obesity after gastrectomy.

In this study, patients with obesity had lower amounts of AAAs following bariatric surgery than they had before the procedure. Liu et al. found that (30) dysbiosis of the gut flora in patients with obesity was linked to an enhanced tyrosine production pathway and raised tyrosine levels. According to cohort research (31), patients’ serum concentrations of AAAs decreased as a result of increased gut microbial activity and higher excretion following weight loss. Lee G et al. (27) have suggested that the decrease in tyrosine concentration after LSG may be related to changes in gut flora and normalization of metabolic imbalances caused by obesity. Tryptophan decreases may stem from restricted intake (32). To identify the mechanism underlying amino acid imbalance, future research could examine the relationship between alterations in gut microbes and amino acid concentrations following bariatric surgery, as this study did not involve the detection of gut microorganisms. Increased serine/glycine concentrations likely reflect improved insulin resistance and altered metabolism (33).

### 4.3 Correlation analysis between amino acid concentration changes and post-LSG alopecia

Bariatric metabolic surgery can help morbidly patients with obesity lose weight and improve metabolic syndrome, but due to changes in the structure and function of the digestive tract, it is highly likely to cause insufficient intake or impaired absorption of nutrients, which can lead to conditions such as alopecia, anemia, or Wernicke’s encephalopathy; therefore, regular monitoring of the patients and appropriate interventions are essential to avoid postoperative complications (34). Alopecia is a common complication after weight loss surgery. In this study, the rate of alopecia in patients who underwent bariatric surgery was 64.18%. The mean time of development of alopecia in the MS group was 2.88 ± 0.87 months after surgery. A meta-analysis (35) discovered that hair loss occurs in 57% of patients following surgery, and while hair loss does not cause major health problems, it does have an impact on patients’ mental health and quality of life. The clinical explanation for the cause of postoperative alopecia is unclear, but it has been proposed that oxidative stress caused by bariatric surgery may inhibit early hair growth, causing the hair follicle to atrophy and enter a resting phase, followed by alopecia lasting about 3 months (36).

### 4.4 Potential mechanisms linking amino acid changes and post-LSG alopecia

Our analysis identified elevated 3-month postoperative leucine as an independent risk factor for alopecia (OR = 1.119, *p* = 0.038). While the precise mechanisms require further investigation, existing literature provides relevant insights. Clinical data demonstrate that androgenetic alopecia patients exhibit significantly reduced plasma zinc levels correlating with decreased follicular density (37). Animal studies further indicate that leucine supplementation can reduce hepatic zinc concentrations, High leucine leads to reduced Zn uptake and Zn deficiency enhances oxidative stress, the latter of which further promotes leucine catabolism and ultimately exacerbates hair follicle damage (38). Kim MK et al. (39) investigated the differences in gene expression of dermal papilla cells (DPs), which regulate hair growth in balding and non-balding areas, in patients with androgenetic alopecia and discovered that leucine-rich repeat containing 15 (LRRC15) was overexpressed in balding-area DP cells compared to non-balding-area DPs, inhibiting cell growth. Leucine is a BCAA and a study by Vincent P et al. (40) discovered that branched-chain amino acid metabolism is disrupted in the body due to diabetes mellitus, metabolic syndrome, and heart failure, resulting in higher BCAAs in plasma. The non-significant elevation of isoleucine and valine may be related to small sample size and individual differences.

This study has several limitations. First, we did not systematically collect data on patients’ family history of alopecia or psychological factors such as stress and anxiety, which may influence hair loss and act as unmeasured confounders. Second, although age differences between the alopecia and non-alopecia groups were not statistically significant, age may still serve as a potential confounding variable affecting both hair loss susceptibility and amino acid metabolism. Third, the relatively small sample size and single-center design limit the generalizability of our findings, and individual variability in obesity-related metabolism and amino acid regulation may affect the interpretation of specific results. Finally, the follow-up period was limited to 3 months postoperatively, which may not fully reflect long-term trends in amino acid changes or hair outcomes. Future studies with larger cohorts, longer follow-up, and more comprehensive data collection are needed to validate these preliminary findings and better understand the underlying mechanisms.

In conclusion, this study suggests that leucine concentration at 3 months postoperatively is an independent risk factor for postoperative alopecia in patients by comparing the serum amino acid concentration in the MS group with the NM group after bariatric surgery. Based on these findings, monitoring leucine levels in the early postoperative period could help identify patients at risk of alopecia. Early detection would allow for timely nutritional assessment and personalized dietary interventions to prevent or mitigate hair loss. Furthermore, increasing awareness among clinicians about the nutritional factors contributing to postoperative alopecia could enhance patient counseling and management strategies.

## Data Availability

The original contributions presented in the study are included in the article/Supplementary material, further inquiries can be directed to the corresponding authors.

## Footnotes

^1^ http://www.chictr.org.cn/
