# Supplementary material for: Correlation between changes of amino acid spectrum and alopecia in patients with obesity undergoing bariatric surgery: a prospective cohort study
Source: Front Nutr. 2025 Aug 26;12:1618630. doi: 10.3389/fnut.2025.1618630 (PMC12417134; doi:10.3389/fnut.2025.1618630)
Supplement: Supplementary file 1 [file Table_1.docx]

Supplementary Material

# Supplementary Tables1. Compasison of baseline plasma amino acid concentration between two groups(μg/ml)

| Amino Acids | MS group | NM group | *p*-Value |
| --- | --- | --- | --- |
| Taurine | 12.06(9.38,17.16) | 15.01(9.92,22.78) | 0.399 |
| Serine | 12.95(11.08,15.75) | 12.55(10.81,15.22) | 0.984 |
| Glutamine | 58.09(49.39,64.70) | 57.39(44.70,62.09) | 0.831 |
| Glycine | 11.80(7.71,15.90) | 12.28(10.12,15.66) | 0.255 |
| Threonine | 10.10(8.42,12.34) | 9.26(7.71,11.50) | 0.552 |
| Aspartic acid | 3.88(3.01,5.44) | 4.85(3.69,6.80) | 0.086 |
| Argnine | 20.05(17.25,25.18) | 17.48(13.52,26.10) | 0.568 |
| Alanine | 27.78(23.69,39.73) | 31.45(23.27,37.00) | 0.761 |
| Glutamic acid | 11.23(9.43,14.07) | 12.73(10.93,16.92) | 0.269 |
| GABA | 1.99(1.18,2.41) | 1.33(0.56,2.53) | 0.358 |
| Tyrosine | 7.99(6.30,9.28) | 7.86(6.44,10.21) | 0.542 |
| Valine | 14.90(11.40,17.04) | 17.36(11.21,20.26) | 0.313 |
| phenylalanine | 6.04(4.67,7.91) | 5.65(4.42,7.72) | 0.619 |
| Isoleucine | 3.13(2.10,3.72) | 3.95(2.14,4.50) | 0.207 |
| Leucine | 10.53(8.60,13.62) | 11.81(7.64,16.81) | 0.726 |
| Methionine | 0.48(0.41,0.50) | 0.48(0.28,0.78) | 0.395 |
| Tryptophan | 1.86(1.20,3.25) | 2.10(1.18,3.03) | 0.811 |

NM: non-mild alopecia; MS: moderate-severe alopecia.

# Supplementary Tables2. Postoperative plasma amino acid concentration of participants (μg/ml)

| Amino Acids | Pre (n=67) | T1 (n=67) | T3 (n=67) | *p*-Value |
| --- | --- | --- | --- | --- |
| Taurine | 13.41(9.79, 19.97) | 13.67(7.24, 23.99) | 18.37(9.32, 25.27) | 0.234 |
| Serine | 12.68(11.05,15.65) | 13.89(11.35,16.56) | 15.09(11.62,18.56)* | **0.019** |
| Glutamine | 57.74(47.78,63.39) | 51.83(43.13,63.83) | 56.87(48.17,68.52) | 0.343 |
| Glycine | 12.29(8.19,15.72) | 19.28(11.69,24.70)*** | 17.83(11.33,25.54)** | **＜0.001** |
| Threonine | 9.82(8.35,12.24) | 8.70(7.08,11.36)* | 10.10(7.71,12.62) | **0.031** |
| Aspartic acid | 4.18(3.30,5.83) | 3.30(2.38,4.27) | 3.69(2.72,6.02) | 0.374 |
| Argnine | 19.70(15.85,26.11) | 14.92(11.54,18.65)*** | 19.11(14.92,23.54)## | **＜0.001** |
| Alanine | 28.56(23.61,37.68) | 20.96(15.78,26.78)*** | 23.69(18.66,32.08)**# | **＜0.001** |
| Glutamic acid | 11.90(9.69,14.45) | 9.28(6.96,11.00)*** | 8.98(6.74,11.53)*** | **＜0.001** |
| GABA | 1.81(0.89,2.41) | 1.33(0.90,1.75) | 1.22(0.94,1.81) | 0.2 |
| Tyrosine | 7.86(6.38,9.49) | 5.01(3.59,6.96)*** | 4.34(3.00,6.91)*** | **＜0.001** |
| Valine | 15.81(11.47,18.32) | 10.83(7.67,16.28)** | 12.10(7.05,16.69)* | **0.007** |
| phenylalanine | 5.99(4.64,7.80) | 4.97(3.21,7.26) | 4.25(2.80,6.24)** | **0.001** |
| Isoleucine | 3.26(2.14,4.11) | 2.18(1.91,4.49) | 2.34(1.43,3.85)* | **0.023** |
| Leucine | 10.96(8.57,14.12) | 10.63(6.31,14.29) | 8.64(5.06,12.96)* | **0.037** |
| Methionine | 0.48(0.32,0.53) | 0.48(0.24,0.61) | 0.48(0.24,0.64) | 0.622 |
| Tryptophan | 1.94(1.21,3.13) | 1.24(0.83,1.93)* | 1.78(0.86,2.48) | **0.021** |

*p*-Value: GEE analysis, to assess whether there were any significant differences caused by the three time.

Pre: preoperative; T1: One month after surgery; T3: 3 months after surgery

(* and bold values)statistical difference between pre and after surgery: **p*＜0.05, ** *p*＜0.01, *** *p*＜0.001；

(#)statistical difference wtih one mouth postopratively. #*p*＜0.05, ## *p*＜0.01.
